# Eco-evolutionary dynamics in response to selection on life-history

**DOI:** 10.1111/ele.12107

**Published:** 2013-04-08

**Authors:** Tom C Cameron, Daniel O'Sullivan, Alan Reynolds, Stuart B Piertney, Tim G Benton, Gabriele Sorci

**Affiliations:** 1 Ecology & Evolution research group, Institute of Integrative & Comparative Biology, University of LeedsLeeds, LS2 9JT, UK; 2 Department of Ecology and Environmental Science, Umeå UniversitySE- 901 87, Umeå, Sweden; 3 Institute of Biological and Environmental Sciences, University of AberdeenAberdeen, AB24 2TZ, UK

**Keywords:** Consumer resource, eco-evolution, environmental change, evolutionary rescue, harvesting induced evolution, life-history, phenotypic evolution

## Abstract

Understanding the consequences of environmental change on ecological and evolutionary dynamics is inherently problematic because of the complex interplay between them. Using invertebrates in microcosms, we characterise phenotypic, population and evolutionary dynamics before, during and after exposure to a novel environment and harvesting over 20 generations. We demonstrate an evolved change in life-history traits (the age- and size-at-maturity, and survival to maturity) in response to selection caused by environmental change (wild to laboratory) and to harvesting (juvenile or adult). Life-history evolution, which drives changes in population growth rate and thus population dynamics, includes an increase in age-to-maturity of 76% (from 12.5 to 22 days) in the unharvested populations as they adapt to the new environment. Evolutionary responses to harvesting are outweighed by the response to environmental change (∼ 1.4 vs. 4% change in age-at-maturity per generation). The adaptive response to environmental change converts a negative population growth trajectory into a positive one: an example of evolutionary rescue.

## Introduction

Our poor understanding of the complex interplay between ecological and evolutionary dynamics hampers our ability to assess the likely demographic and population dynamic consequences of environmental changes (Chevin *et al*. 2010) and the knock-on consequences this will have on ecosystem services and function. This is despite a solid conceptual understanding of the way in which ecological and evolutionary dynamics can be linked (Carroll *et al*. 2007; Kokko & Lopez-Sepulcre 2007; Post & Palkovacs 2009; Schoener 2011). For example, any process that changes population density will likely prompt phenotypic responses affecting demographic processes (e.g. harvesting reduces population size and surviving females increase egg production). Such phenotypic responses can compensate for, or exacerbate, the direct effect on population size (Cameron & Benton 2004; Plaistow & Benton 2009; Schroder *et al*. 2009). In parallel, changes in population structure can provoke evolutionary responses in phenotypes mediated through the deterministic processes of natural or sexual selection (Coulson & Tuljapurkar 2008), or stochastic processes such as drift or mutation (Glinka *et al*. 2003; Nielsen *et al*. 2005; Coulson & Tuljapurkar 2008). Evolved changes in life-histories as a result of both deterministic and stochastic evolutionary processes are likely to affect population structure (e.g. increased age-at-maturity can increase juvenile to adult ratio) and dynamics which will have knock-on consequence for levels of genetic diversity and evolutionary potential. However, teasing apart ecological and evolutionary change in either natural or experimental systems has proven problematic. Although several studies have partitioned the relative importance of evolution vs. ecological mechanisms in life-history change, for example, in changes in offspring investment, phenotype or body size (Hairston *et al*. 2005; Ozgul *et al*. 2009; Coulson *et al*. 2011), there are few empirical studies where evolution affects ecological dynamics at the population or community scale (e.g. Becks *et al*. 2012; Walsh *et al*. 2012). Thus, there is an important missing link in our understanding: for a given system how does environmental change propagate through both ecological and evolutionary mechanisms, and what is the relative speed and strength of responses through each route? This knowledge is essential for any capacity to predict species responses over ecological or evolutionary timescales (Pelletier *et al*. 2009).

Despite the identified need to bring evolutionary biology into population management this is, as yet, rare. There are a number of reasons for this, one of which is the debate on whether evolutionary considerations are of any practical importance to current ecological problems and whether ecological and evolutionary timescales overlap sufficiently to cause interactions. This is most apparent in harvesting literature examining the relative importance of total mortality vs. natural selection caused by selective harvesting in fisheries (Browman *et al*. 2008; Kuparinen & Merila 2008; Andersen & Brander 2009; Kinnison *et al*. 2009). Some argue that evolution of growth rates has not been adequately demonstrated in fisheries (Browman *et al*. 2008), some suggest that while evolution of growth rates has been demonstrated its effects on population biomass are weak (Andersen & Brander 2009), while others suggest that to ignore evolutionary considerations in fisheries is short sighted (Heino & Dieckmann 2008; Kuparinen & Merila 2008; Kinnison *et al*. 2009). Another reason for evolutionary biology not becoming mainstreamed into population management is that it is rarely possible to study genetics, life-histories and population dynamics simultaneously and in sufficient detail, leading to often complex lines of inference to reconstruct unmeasured variables (Coulson & Tuljapurkar 2008; Andersen & Brander 2009; Bonenfant *et al*. 2009; Darimont *et al*. 2009; Ozgul *et al*. 2009, 2012; Coulson *et al*. 2010; Morrissey *et al*. 2012). There is therefore a dearth of examples where the relationship between these components, and the magnitude of their influences, are worked out. Teasing apart the relative contributions would indicate the extent to which changing population density and structure, by selective harvesting for example, results in an evolved response (Coltman *et al*. 2003; Law 2007; Browman *et al*. 2008; Heino & Dieckmann 2008; Andersen & Brander 2009; Kinnison *et al*. 2009). Equally, given environmental change, the role of selection in mediating population dynamics and extinction risks is still unclear (Stockwell *et al*. 2003; Bradshaw & Holzapfel 2006; Chevin *et al*. 2010). Although several studies have documented adaptive phenotypic change in a climate change context, such as reproductive timing in birds (Nussey *et al*. 2005; Charmantier *et al*. 2008), few have considered adaptation empirically under controlled environmental change (Agashe 2009; Willi & Hoffmann 2009; Agashe *et al*. 2011), and fewer still in populations with high standing genetic variation (Bell & Gonzalez 2009, 2011).

Our aim is to use an empirical model system, the soil mite [*Sancassania berlesei* (Michael)], in microcosm to simultaneously characterise life-history, population dynamics and population genetic responses to environmental change. By collecting mites from the field and culturing them in closed populations in the laboratory, we exposed the animals to a radically novel environment. Simultaneously, we compare the relative importance of harvesting through perturbation of population structure by harvesting either adults or juveniles. Over approximately 20 generations we assessed population size and stage structure, while simultaneously assaying population genetic (AFLP diversity and divergence) and phenotypic (age-at, size-at and survival to maturity) changes. This provides the data by which we can dissect the eco-evolutionary relationship between environment, life-history and dynamics through (1) documenting changes in population size and structure, (2) determining the selection upon, and trade-offs between, mean life-history trait values at maturation, (3) identifying the signatures of stochastic and deterministic microevolutionary processes operating within populations and (4) demonstrating how the evolved changes in the life-history in response to environmental change and harvesting contribute to observed trends in population growth and variance. We can therefore document the interplay between ecology and evolution in free-running populations of a sexually reproducing organism with a complex life-history.

## Methods

### Population experiments and harvesting

Wild soil mites were collected from four UK locations and 50–100 mites from each were reared for one generation in excess food then mixed together for a further generation. Eighteen glass tubes (25 mmØ, 50 mm tall, filter paper seal and press on lid) half-filled with standardised density calcium sulphate were selected for microcosms. Each tube was inoculated with ∼ 150 adults of each sex and ∼ 1000 juveniles on day 1 of week 1.

Food was assigned over a 28-day period where each tube received two 0.0015 g balls of dried active yeast per day on average, but in the following repeating pattern: 9 days zero balls, 3 days one, 2 days three, 9 days four, 3 days three and 2 days one ball. The rationale for this periodic food treatment was to generate seasonality of the order of a generation time (∼ 5 weeks) (Ozgul *et al*. 2012). Each tube received at least two drops of distilled water per day to maintain high humidity. Six tubes were randomly assigned to each of three different experimental harvesting treatments: (1) No harvesting; (2) juvenile harvesting, where 40% of juveniles were removed from the population per week; or (3) adult harvesting, where 40% of Adults were removed from the population per week. Using an individual based model (Benton 2012), we estimated that this harvest rate was near maximum sustainable yield.

The number of eggs, juveniles (all stages combined) and adults of each sex in each tube were counted weekly between February 2006 and January 2008 (102 weeks). Unharvested and adult harvested tubes were counted on the same day (Thursday) and juvenile harvested tubes counted the day after. Tubes were counted in the morning, harvesting involved removing the required number of individuals using a fine brush. Harvesting began on week 13 and continued until week 83. Following cessation of harvesting, the dynamics were monitored for a further 18 weeks. Harvesting is stage based, not sized-based, although for a short period following maturation to adulthood (∼ 24 h), while individuals take on an adult physiological state they can still superficially resemble juveniles and may therefore escape harvest. All sizes of juveniles are susceptible to harvesting but smaller juveniles are far more abundant that larger ones. There are four to five harvesting events per mite generation.

### Life-history assays

**Figure 1:**
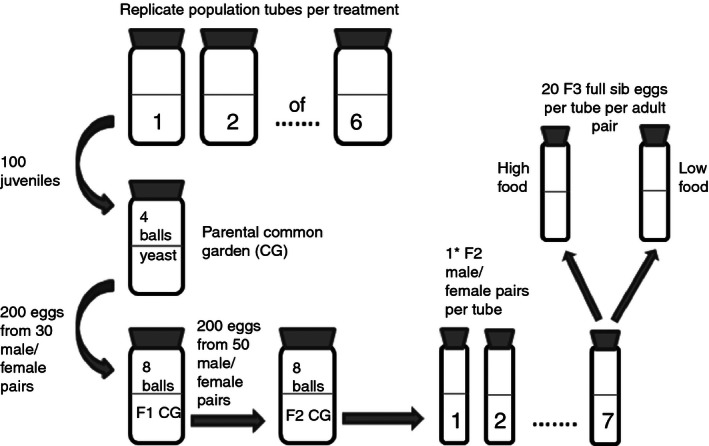
Schematic representation of the assay of life-history to maturation of full-sib families derived from the same two replicates of the six population tubes in each treatment following a common garden rearing environment as used throughout the study. See Box 1.

To evaluate the genetic (non-plastic) responses of the life-history of harvested and un-harvested populations over time, phenotypic assays were conducted following two generations within a common garden rearing environment. We examined age, size, survival to maturity under two different juvenile growth conditions, high and low food, in the 3rd generation following removal from the population (i.e. F_3_) (See Fig. 1 and Box 1).

### Genetic diversity assays

At weeks 0, 18, 37, 63 and 95 females (*n* = 24) were randomly selected from three replicates for each harvesting regime to characterise genome wide genetic diversity using an amplified fragment length polymorphisms (AFLP) assay. Genomic DNA was extracted from individuals using the Wizard DNA extraction kit (Promega Ltd, Madisson, WI, USA) according to the manufacturer's protocol. DNA quantity was assessed using a NanoDrop ND-1000 spectrophotometer (Thermo Scientific, Wilmington, DE, USA) then individuals were AFLP genotyped at five +3 AFLP primer combinations (Eco + CTC − Mse + CGA; Eco + CAG − Mse + CGA; Eco + CAG − Mse + CAA; Eco + ACG – Mse + ATC; Eco + ACG – Mse + AGT) according to the protocols given in Wilding *et al*. (2001). Individuals were scored manually for band presence or absence for all bands between 100 and 350 base pairs observed across all individuals from the wild-type population that gave consistent band intensity greater than 20% of the gel mean. Negative controls were included in each PCR and genotyping step, and a positive control sample was included on every gel.

Analyses of molecular variance (amova) calculated using Arlequin Version 3.5 (Excoffier & Lischer 2010) were used to examine how AFLP diversity was partitioned across treatments, time-points and replicates, and as such identify the signatures of selection and drift operating through the course of the experiment. Specifically, two separate amova hierarchies were defined and examined: First, variance attributable to differences among individuals within replicates, among replicates within treatment groups, and among treatment groups, all within a time-point. It is expected that genetic drift would cause genetic differences to accrue between replicates within treatments, whereas selection would predict significant treatment effects acting either in isolation (with no among replicate within treatment differences) or in addition (significant between replicate within treatment and between treatment effects). Second, differences among individuals within replicates, among replicates within time-points, and among time-points for each of the three harvesting regimes. In this case, it is expected that drift would cause a significant difference among replicates within time-points for each treatment, whereas selection would cause no differences among replicates within time-points, but significant differences across time-points.

AFLP loci that displayed a greater than expected level of genetic divergence between time-points than expected under neutral theory (so-called outlier loci under the effects of directional selection) were identified for each of the three harvesting regimes according to Gagnaire *et al*. (2009) using Dfdist software implementing the hierarchical Bayesian approach of Beaumont & Balding (2004). These loci were then removed from the overall AFLP data set and the amova analyses described above were repeated on what is therefore the putatively neutral component of the AFLP polymorphisms. It is expected that any signature of selection would be removed such that the among-treatment effects in the first hierarchy, and the among-time-point effects in the second hierarchy would be negligible.

To investigate the potential function of any identified AFLP outlier locus, those bands identified as representing outlier loci were excised directly from acrylamide gels and placed in 20 μL of sterile water for 3 h. A 3 μL aliquot was ligated into a pGEM vector (Promega Ltd, Madisson, WI, USA) then transformed into DM103 competent cells, which were plated out on LB agar plates containing ampicillin. Amplicon containing clones were sequenced bidirectionally on an Applied Biosystems ABI3730 automated DNA sequencer (Life Technologies, Carlsbad, CA, USA) according to manufacturer's instructions using standard M13 primers (Sigma-Aldrich Ltd, Dorset, UK). After removal of the vector sequences, a BLAST search was undertaken through the standard NCBI portal.

### Population dynamics and life-history assay analysis

Statistical analysis was conducted in R 2.14.1 (R: A Language and Environment for Statistical Computing, R Core Team, 2012). The significance of temporal trends and harvesting treatments on log transformed population time series were estimated with linear mixed effects models (LME), with repeated measures nested within population tube as a random effect. Model structures and test results are presented in the text. The analyses of life-history changes have been tackled in several ways. Changes in the mean or variance of age or size at maturity over the course of the experiment was estimated with generalised linear models, as was the average daily survival rate per family per treatment. The relationship between age-at-maturity and peak fecundity per female was estimated by linear regression. The effect size of harvesting treatments on the mean phenotype, the age-and-size-at-maturity of each family, at the end of the study was predicted from a manova to jointly model log(age) and log(size) as a function of assay food environment (High or Low food), while controlling for population density in the tube by using covariates [weighted density, median density and total tube survival, see Supporting Information (b)]. Data on age (days), size (mm) and survival (proportions) at maturity are presented as full-sib female or treatment means (e.g. Plaistow *et al*. 2004).

To compare the effects of changing life-histories on population dynamics, we estimated the instantaneous rate of reproduction (*R*_0_) at each life-history assay time point using data from the low-food assays. The low food life-history data are used to estimate *R*_0_ as resource limitation regulates population size, so the low-food assays are particularly indicative of microcosm population conditions. *R*_0_ was estimated from the sum of survival probabilities to maturity times lifetime reproduction, controlling for generation time (see Supporting Information). Mean and confidence intervals of *R*_0_ are generated by resampling from the distribution of life-history trait values specific to each assay time point within treatment groups. As *R*_0_ summarises the life-history data underlying population processes, it is also a measure of population growth rate (when *R*_0_ > 1 the population is growing and when *R*_0_ < 1 the population is shrinking). To check that the life-history based estimate of population growth corresponds to population patterns, the average population growth rate (*pgr* = Nt + 1/Nt) was calculated from a smoother fitted across replicate population *pgr* time series per treatment, and a correlation test between the two estimates of population growth undertaken.

**Box 1 Estimating changes in life-history through ecological time**

Life-history assays were conducted on the progeny of females collected from the same two of six replicate experimental populations per harvest treatment (Fig. 1). For the common garden, 100 small juveniles were removed from the two replicate populations per treatment and reared until mature. Then 30 male–female pairs were mated collectively and 200 of their eggs were transferred to a new tube and reared on *ad lib*. food until mature. 50 male–female pairs of these *F*_1_ progeny were mated collectively and 200 of their eggs transferred to a new tube and reared on *ad lib*. food. Using a common garden approach for three generations minimises the often strong confounding influence of maternal environments on the results of life-history experiments (Plaistow *et al*. 2006). Ten newly matured male and female mites were selected from each *F*_2_ population line at random and placed in assay tubes (Flat base cylinder 50 × 16 mm 7 mL, PS with 17 mm pierced push stop lid, with trapped 25 mm diameter filter paper, half-filled with plaster) with a single 1 mm rod of commercial baking yeast (∼ 0.3 mm dia). After 24 h the pair was placed in a new tube with a new rod of yeast and the initial eggs discarded to avoid eggs that may have been fertilised by other males. After a further 24 h period, 60 eggs were collected from seven of the ten pairs (*n* = 10 in wild assay). For each male–female pair, 20 of their eggs were placed in one of two new assay tubes. One to be fed High food (10 μL air-dried flake from 0.5 g baking yeast dissolved in 10 mL distilled water = 0.5 mg day^1^) and one to be fed Low food (0.3 μL air-dried flake supplied every second day = 0.0075 mg day^1^) *per capita* daily food. During the initial period of the assay when mites were very small, the high food flake would last several days before needing to be replaced. The original egg collection tube is kept without the adults from each pair but containing any remaining eggs, on day three after laying if any eggs remain unhatched in the assay tubes they are replaced with same aged hatchlings before feeding commences to maintain initial density. Individuals in assay tubes were counted, fed and watered daily and when mature male and female individuals were seen, they were photographed for later measurement using a Nikon Digital Sight 5.0 megapixel camera (DS-5M) attached to a Nikon SMZ1500 stereomicroscope under × 40 magnification, then removed (Nikon Instruments UK, Surrey, UK).

The first assay was on the ‘wild derived’ population (control) used to set up the replicate population experiments at time zero (24th March 2006, following the three generation common garden) and then at weeks 18, 37, 63 and 95. We thus assayed seven families, from each of two (from six) replicate populations, in high and low-food conditions, for three harvesting treatments at five time points (420 family assays). From these assays, we obtained the family mean and variance in age and size at maturation and survival to maturation for adult females. As the tubes were monitored, fed and watered daily we were able to monitor daily survival rates and density changes to use as covariates in later analysis.

Data from a previous experiment were used to estimate the effects of juvenile rearing environment on female fecundity (Plaistow *et al*. 2006). Juvenile mites were selected from stock and reared on Low juvenile food for three generations (*F*_3_). Following maturation in the third generation, females were placed with two males from stock and monitored daily for eggs on a fixed food quota until day 7 post eclosion. Female age and size at maturity was also recorded.

## Results

### Population dynamics

**Figure 2:**
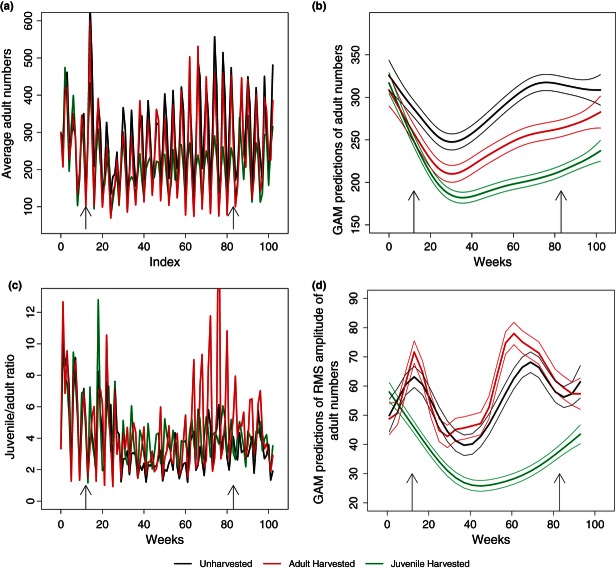
Adult numbers shown as; (a) spline smoother fitted to the adult numbers, averaged across replicate populations per week and (b) predictions (± 95% CI) of total average adult population size from GAM fit across all weekly counts per treatment (4 d.f.). (c) Juvenile:Adult ratio calculated from the averaged counts. (d) GAM fit to Root Mean Square amplitude of adult numbers in each replicate population as a measure of variance (mean ± 2 SE) where RMS is calculated across all 28 day periods (3 d.f.). Arrows on x-axis indicate when harvest starts in week 13 and ends week 83. Degrees of freedom for GAM models selected by stepwise AIC model simplification.

The population dynamics changed markedly over the course of the experiment (Fig. 2). The initial response, common across all treatments and life-history stages, was a marked decline in population size (∼ 50% of total population size). This decline lasted for approximately 30 weeks (∼ 6 generations, Fig. 2a–c). Subsequently, there was an increase in mean population size of all life-history stages, which was also common across all treatments but the magnitude of which was differentiated by harvesting (Fig. 2a–c).

In the harvested treatments, the final population size is reduced relative to the unharvested treatments, such that adult harvested treatments lead to adult population sizes about 86% of the unharvested (Fig. 2b), and juvenile harvested treatments having adult population sizes of about 70% of the unharvested populations (Fig. 2). The stage structure also changes over time with clear shifts in the juvenile:adult ratio [Fig. 2c, Supporting Information (3a)]. Harvesting juveniles dampens, and harvesting adults increases, variability in densities in response to forced seasonality in resources; although the magnitude of these effects varies temporally (Fig. 2d, GLM: variance in adult numbers ∼ time period × treatment, tube nested within treatment, interaction *F*_6,15_ = 6.12, *P* < 0.0005). Following release from harvesting (week 83), there is an ecological response such that mean and variances between treatments become more similar, but significant differences associated with harvest treatment remain to the experiment's end (Weeks 83–102, LME: log(adult population) ∼ harvest treatment, *F*_2,15_ = 10.2, *P* < 0.002; log(juvenile population) ∼ harvest treatment, *F*_2,15_ = 3.724, *P* < 0.05; adult population variance ∼ harvest treatment, *F*_2,15_ = 4.26, *P* < 0.04).

### Phenotypic dynamics

**Figure 3:**
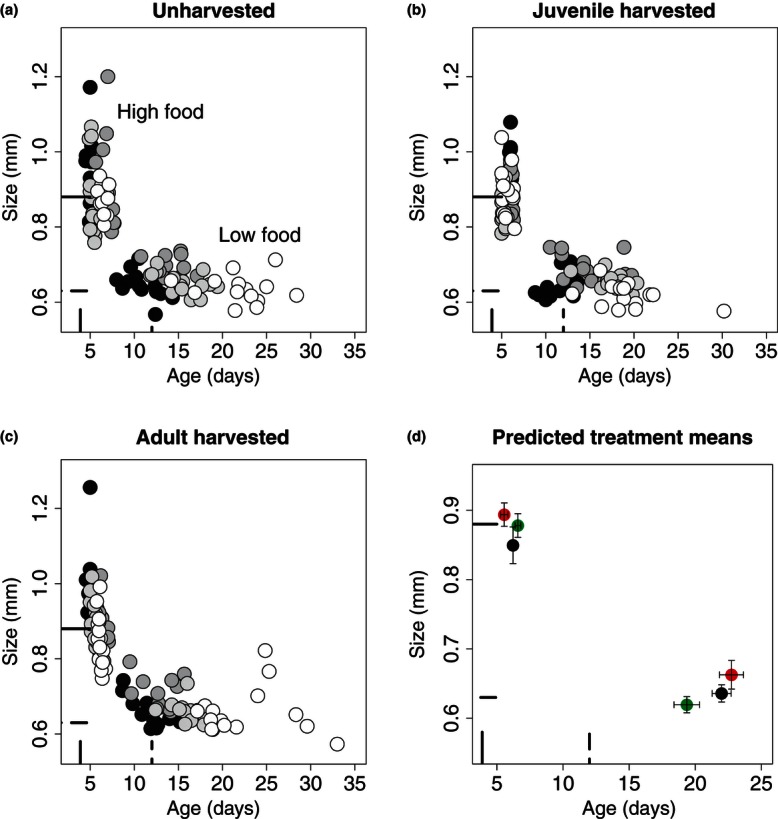
Age and size at maturity. Each circle (a–c) represents the full-sib female means under each juvenile growth condition of high food (which creates a cloud of points with variation in the vertical axis) and low food (creating variation in horizontal axis, see Box 1 and annotation on fig. 2a) from (a) unharvested, (b) juvenile harvested and (c) adult harvested populations. Symbol shade represents time; black = 18 weeks, dark grey = 37 weeks, light grey = 63 weeks and white = 95 weeks. The mean wild-type phenotype at time zero for all treatments is indicated by bars on the axes (high food: black, low food: dashed). Differences over time and between treatments are significant. Predicted Treatment means (d) at the end of the experiment accounting for differences in mortality caused by different development rates (manova, see text and Supporting Information). Colours are treatments: unharvested (black), juvenile (green) and adult harvested (red).

There are clear changes in the mean phenotype, measured under standard conditions, during the experiment associated with the observed changes in population dynamics (Fig. 3). First, pooling the data from both the high and low growth conditions, as the experiment progresses, overall trait variation increases. For example, for the unharvested treatments, the covariance of age-and-size to maturity across family means doubles from −0.4889 from week 0 to −0.9855 at week 93 (GLM: cov ∼ assay time, *F*_4,14_ = 18.51, *P* < 0.001; Fig. 3). Second, a predominant signal across all treatments is that the age to maturity increases markedly with time (GLM log(age) ∼ time-point × assay food, *R*^2^ = 89%, assay time: *F*_1,488_ = 526.03, *P* < 0.001). This is especially noticeable under low-food conditions, which most closely represent those within the food-limited microcosms, where there is an increase in development time from 12.5 days at the start of the experiment to 22.0 days at week 95 (an increase of 76%) (Fig. 3, see also Table S5). This increase in age to maturity is significantly moderated by harvesting treatment: juvenile harvested treatments mature on average earlier (median 18 days, 20% shorter than the unharvested treatments at week 95 and 1.5 × the initial wild types). On the other hand, adult harvested treatments mature slightly older (median 26 days, 20% longer than the unharvested treatment at week 95 and 2.16 × the initial wild types), and on average at a greater size (0.7 mm cf. 0.62 mm, a 13% increase).

Size-at-maturity, although not as obviously as age-at-maturity, changes significantly both during the experiment and between treatments. Size initially increased, temporally associated with the initial decline in population size, prior to declining during the latter period of harvesting [GLM log(size) ∼ assay time × assay food, *R*^2^ = 87%, assay time: *F*_4,482_ = 16.049_,_ *P* < 0.0001, Fig. 3). This increase-then-decrease pattern is most noticeable in the juvenile harvested treatment under low food (Fig. 3b). Changes also occurred in the age-and-size-at-maturity under high food: age-at-maturity increased from an initial 4–6 days (unharvested and adult harvested) or 5 (juvenile harvested). At week 95, which occurred ∼ 4 generations following the cessation of harvesting (12 weeks in population, plus 2 generations of common garden), the treatment differences remained highly significant [Fig. 3d, Supporting Information (3b), Fig. S2]. With low food, juvenile harvested phenotypes matured earlier than unharvested, and adult harvested matured at about the same time but larger; with high food, juvenile harvested matured earlier and adult harvested smaller than unharvested.

The predominant phenotypic change in low-food conditions under all harvesting treatments is an increase in age-at-maturity. We found no significant change in daily survival rates between treatments or across the study (anova; *F*_5_,_83_ = 0.1545, *P* > 0.9), suggesting no trade-off between slower growth and greater survival, although there is a survival cost in that the overall risk of mortality depends on the time as a juvenile. An analysis of existing experimental data on female performance under similar low-food experimental conditions indicates that the relationship between growth rate and fecundity is positive, such that delayed maturation is linked to higher fecundity [regression, fecundity (days 3–5) = 0.083 × age-at-maturity, *F*_1,10_ = 135.2, *P* < 0.03; *R*^2^=0.92], suggesting that, under low-food conditions, growth rate is traded off against fecundity.

### Linking phenotypic and population dynamics

**Figure 4:**
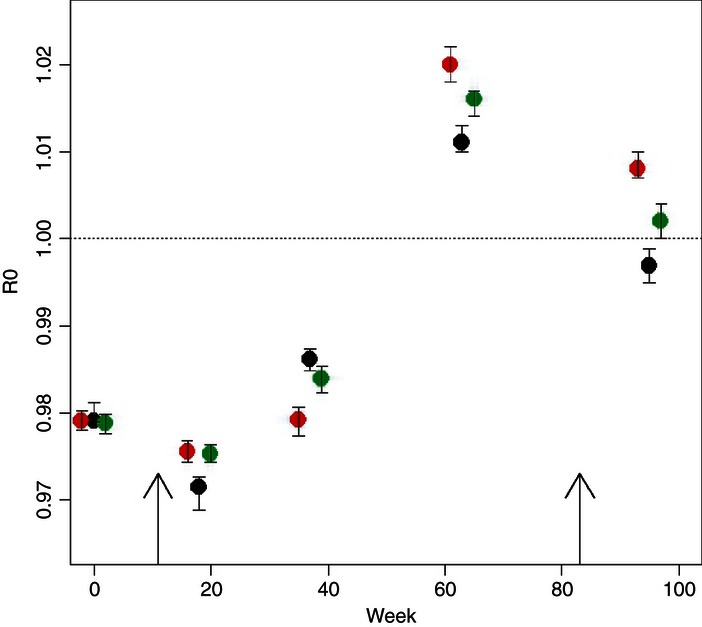
Population growth rates estimated as *R*_0_, the basic reproductive ratio, calculated from life-history assay data at assay time-points (see text and Supporting Information). Colours are treatments: unharvested (black), juvenile (green) and adult harvested (red). Jitter added to data points at each time-point to separate overlapping points. See Supporting Information for detailed methods. Arrows on *x* axis indicate harvest starts in week 13 and ends week 83.

We calculated population growth rate as the basic reproduction rate per generation (*R*_0_), based on phenotypic data from the low-food life-history assay. *R*_0_ is strongly correlated with population growth rate measured directly from the time series (Pearson's correlation = 0.854, *t*_13_ = 5.92, *P* < 0.0001). Initially, population growth rates were less than one, indicating a trajectory towards extinction, with lower growth rates in the harvested treatments (Fig. 4). Evolution of the life-history reversed these declines and *R*_0_ increased to considerably more than one, and following the cessation of harvesting (and the re-imposition of strong density-dependence) both harvested population growth trajectories were closer to 1.0 (week 95). The *R*_0_ of the juvenile harvested and adult harvested treatments were both significantly greater than the unharvested treatments at the end of the experiment as the confidence intervals of their mean do not overlap.

### Patterns of genetic diversity

**Figure 5:**
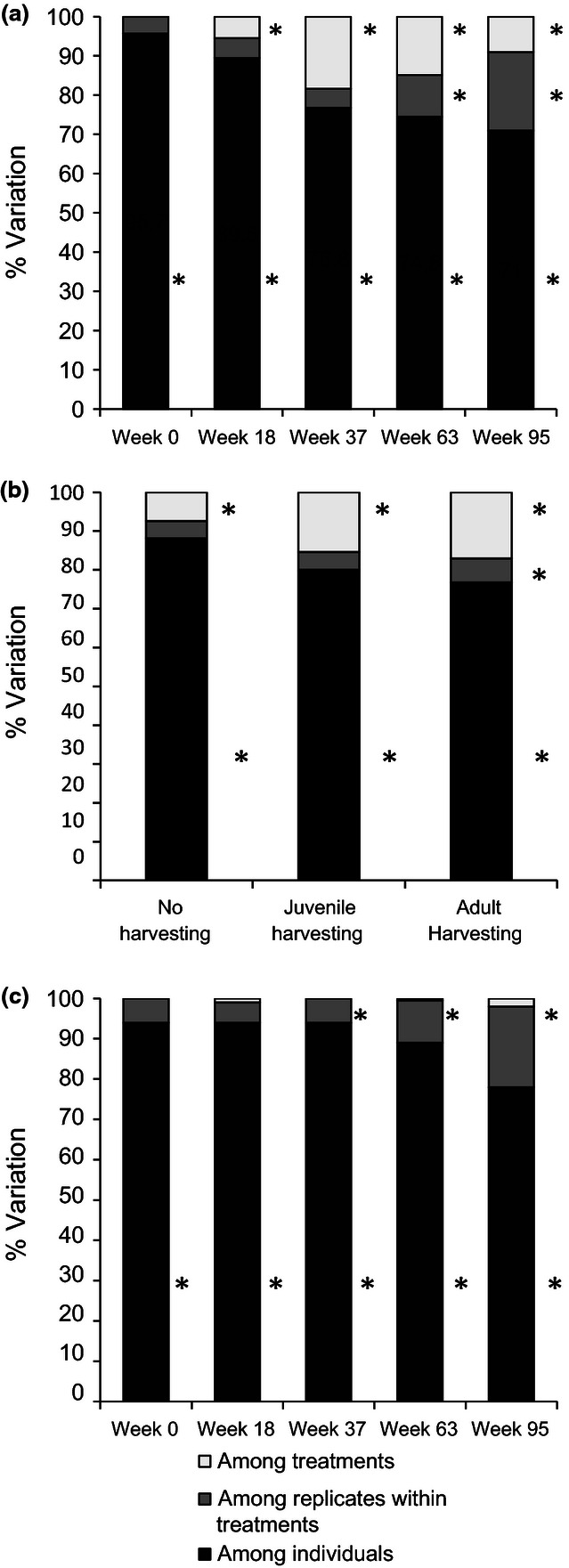
Analyses of molecular variance (amova) illustrating how genetic variation is apportioned: (a) among individuals, among replicates within treatments and among treatments for each sampling time-point across all AFLP loci; (b) among individuals, among replicates within time-points and among time-points for each treatment across all AFLP loci; (c) among individuals, among replicates within treatments and among treatments for each sampling time-point for the putatively neutral AFLP loci (i.e. after removal of all outlier loci). Bar height represents the percentage variation explained, with an asterix indicating a significant component of the overall variance (*P* < 0.05).

Individuals were scored across a suite of 293 AFLP markers identified as polymorphic in the wild-type population (*H*_e_ = 0.24). Marked genetic changes were observed through the course of the experiment consistent with the effects of both selection and drift. There were clear and significant shifts in allele frequencies over time and across the different treatment groups. amova indicated that from week 18 onwards there was a significant effect of treatment on how genetic variation was apportioned among populations (Fig. 5a). This is consistent with the effects of selection given that these are differences above and beyond any within-replicate differences that would have indicated a signature of drift. Indeed, up to week 63, there were no associated differences attributable to variation among replicate populations within treatments, indicating the effects of drift were minimal. There was a gradual reduction in genetic diversity throughout the experiment concomitant with reduced effective population size of populations in microcosm (Lee *et al*. 2011), and as such beyond week 63 there were significant effects of both treatment and replicate within treatment, indicating that genetic drift affects diversity near the end of the experiment, where it may mask larger adaptive responses of the populations. For all three harvesting treatments, there was a significant differences across time-points (Fig. 5b), indicating gradual genetic shifts during the experiment. This was in the absence of any significant effect of differences among replicates for the juvenile harvesting and no harvesting treatments, again indicating the effects of selection. In the adult harvesting group, both drift and selection are operating in tandem to drive allele frequency shifts through the experiment.

Multiple AFLP markers were identified as under the influence of directional selection as they displayed greater genetic divergence than expected under neutral theory. Several such outlier loci were identified across treatments (Fig. S3). Between weeks 0 and 37 when the greatest difference among time-points could be attributed to among-treatment effects (Fig. 5b), ten outlier loci were identified under adult harvesting, seven under juvenile harvesting and seven without harvesting (Fig. S4b). Of these, 3/10 loci under adult harvesting and 2/7 loci under juvenile harvesting were identified in more than one replicate population, indicating these are unlikely false positives. None of the outlier loci were common across harvesting regimes. When the outlier locus band was excised from the gel, cloned into an *E. coli* vector and subsequently Sanger sequenced, none yielded a significant BLAST return (e^−3^) from which function could be intimated. When the amova were repeated on the AFLP dataset after removal of all outlier loci, the significant among-treatment effect for all five time-points was removed (Fig. 5c).

## Discussion

Our data strongly indicate that exposure to a novel environment, and perturbations to stage/age structure created by harvesting create selection on the life-history that alters demographic performance and ultimately population dynamics. The evolutionary response to environmental change occurs rapidly and interacts with the response to the perturbations caused by harvesting – which are indicated by the rapid dynamical changes shown at the cessation of harvesting (Fig. 2a,c).

The initial trajectories of population size were all negative. Using our phenotypic data to estimate *R*_0_ shows that it was initially less than one, indicating a trajectory towards extinction, with lower growth rates in the harvested treatments (Fig. 2, Fig. 4). Evolutionary change in the life-history, as summarised in *R*_0_, converts a negative population growth rate (where *R*_0_ < 1) to a positive one ( *R*_0_ > 1). Following the cessation of harvesting (and the re-imposition of strong density-dependence) *R*_0_ ended up closer to 1.0 (week 95). The estimated *R*_0_ of the juvenile harvested and adult harvested treatments are both significantly greater than the unharvested treatments at the end of the experiment, as are their average population growth rates. In our study, experimental populations adapt to laboratory microcosm conditions, where there is significant density-dependent competition due to food limitation, by increasing fecundity via a trade-off with development rate, while other life-history traits remained unchanged. Other experimental evolution studies have shown a U-shaped recovery from a decline in growth and this has been called evolutionary rescue. Increased fecundity was also found to be the determinant of rescue in a recent study of experimental *Tribolium* populations adapting to a novel resource environment (Agashe 2009; Agashe *et al*. 2011). Several studies have shown that the probability of a population surviving an abrupt environmental change is increased with increasing initial genotypic diversity, either by showing that increased population size is correlated with genotypic diversity (Bell & Gonzalez 2009, 2011; Willi & Hoffmann 2009), or by manipulating genotypic diversity directly (Agashe 2009; Agashe *et al*. 2011). Our study is novel as it follows phenotypic, population genetic and population dynamics simultaneously through a rescue. The rescue occurs in all replicates of all treatments, most likely given the high standing genetic diversity at the onset of the experiment, which is largely retained at week 37 in the experiment. Our experimental treatment involves enclosing a population and creating strong density-dependence, a proxy for many ecological situations involving habitat loss and fragmentation, rather than exposing populations to imposed selection such as heat or toxicity, suggesting that the potential for evolutionary rescue in population management and conservation is real.

We have demonstrated an eco-evolutionary response in single species population dynamics responding to directional change in the environment (wild type to laboratory microcosm resulting in new competitive conditions and reduction of fecundity). The directional environmental change is caused, in this simplified experimental setting, by soil mites competing for reduced *per capita* resources. This creates selection on the life-history which further changes the competitive environment. Clearly in a completely free-running system, the evolving change in mite population structure would create selection on its natural resource populations. Directional change in the environment is most likely under climate change scenarios and it is directional change that is being considered most in field studies of adaptation in a climate change context (Nussey *et al*. 2005; Charmantier *et al*. 2008; Hendry *et al*. 2008; Ozgul *et al*. 2009; Coulson *et al*. 2011). The importance of rapid adaptive phenotypic evolution is only just being appreciated in an environmental change context and predictive ecology requires that we move beyond considering shifting trait means and variance a nuisance and instead try to understand how it can aid eco-evolutionary responses to changes in the local environment (Chevin *et al*. 2010).

We found ecological effects of harvesting, as indicated by changes in population dynamics following the release from harvesting. However, even over the relatively few generations of the experiment, the ecological and evolutionary contributions of harvesting to population dynamics are closely intertwined, and not as traditionally considered separable. Harvesting created greater population differentiation in quantitative traits than neutral markers in a manner consistent with current theory. Unlike classical fisheries size-structured harvesting, where we expect size-structured harvesting to select for reduced size-at-age, (i.e. somatic growth rates) we expect stage-structured mortality (harvesting or natural predation) to select against time spent in vulnerable stages (Reznick *et al*. 1990; Ernande *et al*. 2004). Compared to unharvested treatments, juvenile harvested phenotypes mature earlier, consistent with an adaptive response to escape the life-history stage suffering highest mortality (Ernande *et al*. 2004). Similarly, adult harvested treatments mature slightly later and larger. As they are entering a high-mortality phase, selection for delayed maturity increases instantaneous fecundity, and is therefore also consistent with expectations of an adaptive evolutionary response (Ernande *et al*. 2004). These results are wholly consistent with the classic experimental life-history evolution of wild guppies in tropical streams driven by predation by large or small predators specialising on adult or juvenile guppies (Reznick *et al*. 1990, 1996). Harvesting juveniles therefore dampens population dynamics not only by reducing overcompensatory responses (Cameron & Benton 2004) (the ecological response) but also by reducing individual growth rates to maturity; slowing down compensatory responses to mortality (the eco-evolutionary response). Harvesting adults excites population dynamics by promoting overcompensatory responses (e.g. competitive release, the ecological response Fig. 2a,d), but also by selecting for increased instantaneous fecundity (the eco-evolutionary response).

Our results support the conclusions of a range of other studies on harvested systems that lack the ability to simultaneously track genetic, phenotypic and population changes. Despite the accumulated evidence from microcosm studies on the importance of evolutionary considerations for harvested populations, a number of reports have been critical of microcosm approaches. One such critique is that the rates of evolved trait change in experimental approaches are far greater than that observed in wild exploited populations (Andersen & Brander 2009). However, with approximately 1.4% change in trait mean per generation, the evolved rate of phenotypic change in our study is similar to inferred ‘slow’ rates of evolutionary change in extant fisheries (Andersen & Brander 2009). We therefore robustly confirm the potential for harvesting to cause evolved changes with population dynamic consequences in ‘ecological time’. We would also highlight that the evolutionary response of mite microcosms to environmental change was far greater (4% change per generation) than the response to strong selective mortality.

In conclusion, we identified an eco-evolutionary population response where changes in individual somatic growth rates are caused by natural selection acting on the life-history in response to a combination of increased competition for food and change in imposed mortality. The response to selection leads to changes in individual growth rates, fecundity and population dynamics. In particular, we have witnessed adaptation to local environmental conditions (e.g. changing resource availability) that permitted recovery of a declining free-running population over approximately five generations. Harvesting caused the mean phenotype of harvested populations to deviate from unharvested phenotypes which contributed to long term differences in population growth rates between harvested and unharvested populations. However, due to evolutionary rescue, harvesting yields were 13% higher under adult harvesting at the end of the harvesting period than they would have been had the population been maintained at the density prior to the recovery at around week 40. The rapid eco-evolutionary responses to environmental change observed indicates a potential for evolution to help management aims more than has been normally appreciated.
