# Supplementary material for: Eco-evolutionary dynamics in response to selection on life-history
Source: Ecol Lett. 2013 Apr 8;16(6):754–63. doi: 10.1111/ele.12107 (PMC3712461; doi:10.1111/ele.12107)
Supplement: Supplementary file 1 [file ele0016-0754-SD1.pdf]

Supporting Information for

T.C. Cameron*, D. O'Sullivan, A. Reynolds, S.B. Piertney, T.G. Benton
* To whom correspondence should be addressed.
TCC (tom.cameron@emg.umu.se)

This PDF file includes:

1. Introduction to Supplementary Information
2. Methods
3. Further analyses and supporting graphs
   (a) Population dynamics SI
   (b) Plot of the MANOVA fits vs. the family means to show that the model is a good fit. Explanation of analysis and tables of results
   (c) Outlier analysis for AFLP loci
   (d) Calculation of R_0 values from the life-history data
4. References for SI

1. Introduction to Supplementary Information

This supplementary information (SI) has been provided to give more detail about our study system, methods and analysis. Further analyses include plots of juvenile/adult ratios to see changing demography, plots to show how repeatable replicate time series were and a full statistical model representing the direct and delayed density dependent effects on survival and recruitment. A full explanation of the multivariate approach we took to jointly model age and size at maturity and how this model was used to predict co-dependent age and size at maturity for each assay time point is provided.

We present here in the Supplementary Information two plots to supplement our analysis of molecular variance that describes how a number of our AFLP markers were probably under directional selection and that we found significant levels of population differentiation in quantitative traits. Finally, we present a full description of the methods used to estimate rate of change of population size from life-history that allows us to consider how well information from the life-history assays can predict changes in population growth.

2. Methods

Model System

The soil mite, *Sancassania berlesei* (Michael), is used for the experimentally harvested model system. This species has a plastic life-history in that growth rates, survival and fecundity vary with resource allocation (Beckerman *et al.* 2003). Furthermore, juveniles compete with adults, and are morphologically similar, and the juvenile phase is typically short relative to the adult phase (Plaistow *et al.* 2004). Juveniles can be identified as distinct from adults due to the relative size and shape of different body parts. Adults are competitively dominant over juveniles as they can move to new food patches faster and pull juveniles off existing food patches. But juveniles can feed amongst detritus and underneath adults, and it should be remembered that there are many more juveniles than adults. If small juveniles are culled, maturation rates into the adult class increase suggesting the juveniles are food limited and adults are better competitors (Cameron & Benton 2004). Under equilibrium conditions the population has noisy equilibrium like dynamics. Previously we have shown that the life-history of this organism can respond to changes in current and previous environments and there are high levels of genetic variation in heritable life-history traits (Beckerman *et al.* 2002; Beckerman *et al.* 2006; Plaistow *et al.* 2006). And we have shown that this system lends itself well to investigating the population dynamics consequences of harvesting (Cameron & Benton 2004) and with a short generation time (10-50 days) it is suitable for asking evolutionary questions over contemporary timescales. General laboratory and rearing techniques can be found in work we have published previously (Benton *et al.* 2001).

3. Further Analysis and Supporting Graphs

(a) Population dynamics SI

The dynamics can be summarised as having two main features, an initial decline in mean abundance of all stages that takes approximately 40 weeks (total population, the minimum is different for each stage) and then a second phase where the mean and variance in density increased (Figure S1). In the unharvested control the mean density levels off at the end of the experiment around week 70. The increase in variance and the levelling off is particularly evident in the juvenile and juvenile:adult ratio plots (Figure S1 b).

Using an *a priori* list of candidate models that include recruitment to and survival within each life-history stage, harvesting, time and their interactions models that captured the logged population dynamics of the mites best were chosen based on their weighted AIC values such that we did not assume there was one best model (Table S1). Log transformations were undertaken as a precaution to ensure normality and reduce patterning in the residuals. The best set of models with ΔAIC <6 is presented to estimate the approximate effect of each harvesting treatment on recruitment, survival and population growth of each stage; not as a means to predict population dynamics (Table S2).

The best statistical model of the population dynamics of each stage, including direct and delayed density dependence (where stage densities are influenced by current and past mite stage densities), shows significant changes in the dynamics of all stages over time (Table S1 and S2). However, the sign and magnitude of these changes differ between stages (stage*week interactions, for example: The average coefficients from the set of selected models show that juvenile survival increased over time (Survival:Week coef = 0.0019) whereas adult survival did not change over time (Survival:Week coef = 0.00). This is what leads to the changes in the relationship between juveniles and adults over time (Figure S1b). The increasing amplitude of the cycles in in all stages under all harvesting treatments occurs around week 60 coinciding with the period where juvenile development periods approach the 21 day mark (see LH assay timepoint 3 results, week 63). 21 days represents when mite populations would receive their 3rd harvesting event per generation.

Imposition of harvesting leads to immediate changes in age structure: harvesting adults significantly reduces adult densities via reducing adult survival (i.e. direct lagged effect of harvest, Adult dynamics model: Survival coefficient for adult harvested = -0.11 log scale = 11%) and reduces the density of potential recruits via indirectly reducing juvenile densities (Juvenile dynamics model: Survival coefficient for adult harvested = -0.17 log scale = 16%)(Table S2). So despite removing 40% of adults each week, this change in adult numbers is partially compensated for via a reduction in juvenile densities. This occurs due to a significant negative relationship between juvenile densities and adult recruitment (such that large juvenile densities result in reduces adult recruitment (model averaged coefficient -0.09 (log scale)) partially offsets these direct and indirect negative effects by reducing competition such that recruitment increases leading to rapid replacement of harvested adults.

Post harvesting in unharvested and adult harvested populations, adult numbers become less variable but remain high whereas when released from juvenile harvesting adult numbers increase but their amplitude remains relatively unchanged.

Inter-replicate variability is small (Figure S1d) and where data has been missing due to staff absence at weeks 20, 56, 83 and 97; the data were spline interpolated over weeks 19-21 but due to the nature of interpolations poorly predicting peaks and troughs the average of the two peaks or troughs adjacent to the missing value were used on weeks 56, 83 and 97.

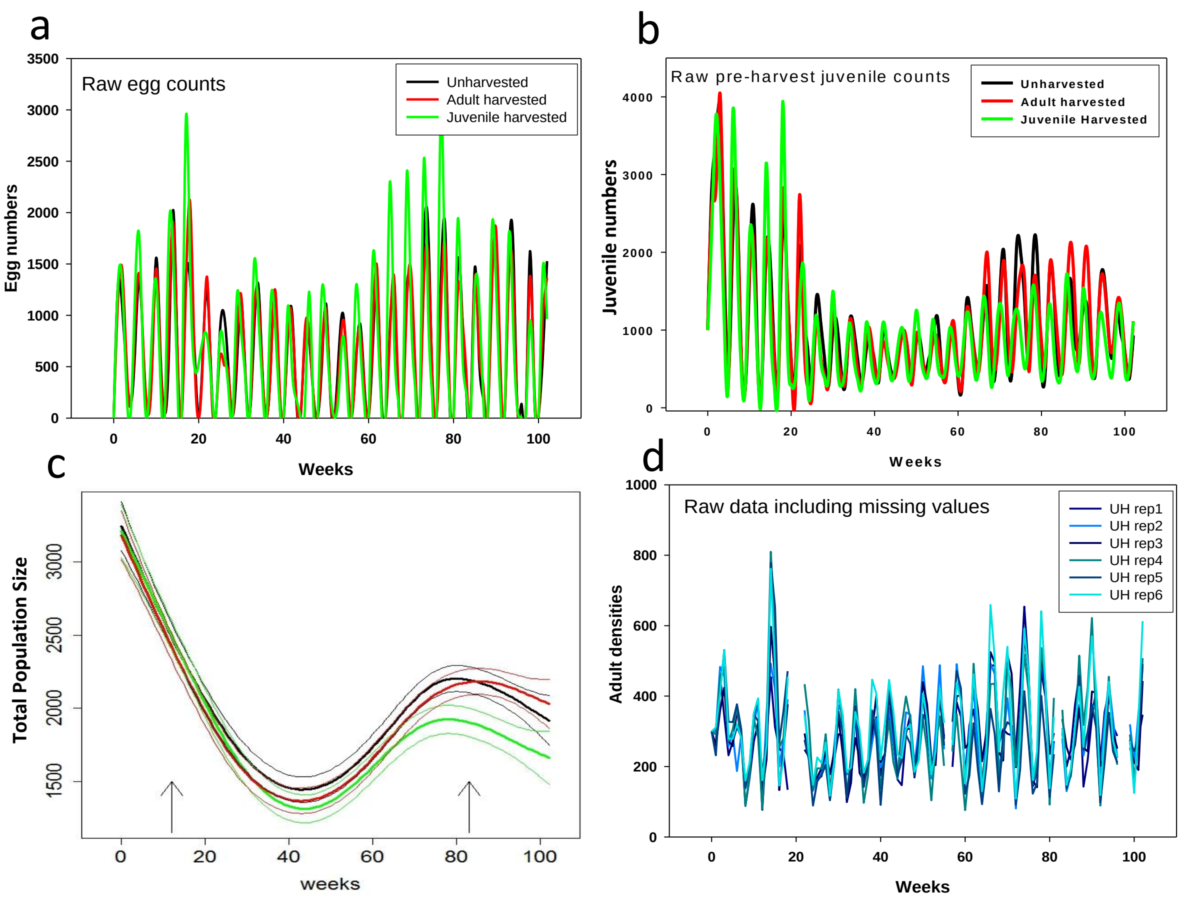

Figure S1. Panel plot showing the average across 6 replicates of the pre-harvest weekly census for (a) juveniles,(b) eggs, (c) Mean total population size fitted using a prediction from a General Additive Model (4 degrees of freedom selected by AIC) and (d) Unharvested adult number data.

Table S1. The table shows the Aikake weight using corrected AIC for the better candidate models for the effects of survival and recruitment per life-history stage and their interaction with harvesting on adult, juvenile and egg population dynamics. Null and alternative models for discussion also included. K refers to the number of estimated parameters including fixed and random effects where Ad, J and E = Adults, Juvenile and Egg densities respectively, t.1 and t.2 = one and two week lag respectively, har = 40% harvest of Adults or Juveniles every 7 days and week is a continuous variable. * indicates the main effects and their interactions.

Adult density **n=1836**

name	Model	AIC	logLik	K	ΔAICc	rank	exp	wi	R^2
Ad.6	log(Adt.1+1)*week*har + log(Et.2+1)*week*har	1739.27	-848.63	21	0.00	1	1.00	0.5227	37.60
Ad.19	log(Adt.1+1)*week + log(Et.2+1)*week*har	1739.45	-852.72	17	0.18	2	0.91	0.4773	37.40
Ad.18	log(Adt.1+1)*week*har + log(Et.2+1)*week	1771.50	-868.75	17	32.23	3	0.00	0	36.29
Ad.5	log(Adt.1+1)*week + log(Et.2+1)*week +har	1784.47	-881.23	11	45.20	4	0.00	0	35.51
Ad.4	log(Adt.1+1)*week + log(Et.2+1)*week	1812.60	-897.30	9	73.34	5	0.00	0	35.91
Ad.3	log(Adt.1+1)*week*har + log(Jt.1+1)*week*har	2262.11	-1110.06	21	522.84	15	0.00	0	22.12
Ad.NULL	~1	2387.42	-1189.71	4	648.16	20	0.00	0	~

Juvenile density **n=1836**

name	Model	AICc	logLik	K	ΔAICc	rank	exp	wi	R^2
J.15	log(Et.2+1)*week*har + log(Et.1+1)*week*har	3085.33	-1521.66	21	0.00	1	1.000	0.9988	35.21
J.19	log(Et.2+1)*week + log(Et.1+1)*week*har	3098.77	-1532.39	17	13.45	2	0.001	0.0012	34.62
J.18	log(Et.2+1)*week*har + log(Et.1+1)*week	3121.77	-1543.88	17	36.44	3	0.000	0	34.26
J.13	log(Et.2+1)*week + log(Et.1+1)*week	3124.44	-1553.22	9	39.11	4	0.000	0	33.1
J.NULL	~1	3852.15	-1922.07	4	766.82	20	0.000	0	~

Egg density **n=1836**

name	Model	AICc	logLik	K	ΔAICc	rank	exp	wi	R^2
E.14	log(Et.2+1)*week + log(Et.1+1)*week +har	7134.48	-3556.24	11	0.00	1	1.000	0.81584	18.45
E.13	log(Et.2+1)*week + log(Et.1+1)*week	7138.95	-3560.48	9	4.47	2	0.107	0.08736	18.26
E.19	log(Et.2+1)*week*har + log(Et.1+1)*week	7139.50	-3552.75	17	5.02	3	0.081	0.06647	18.66
E.18	log(Et.2+1)*week + log(Et.1+1)*week*har	7141.28	-3553.64	17	6.80	4	0.033	0.02727	18.58
E.15	log(Et.2+1)*week*har + log(Et.1+1)*week*har	7145.65	-3551.82	21	11.16	5	0.004	0.00307	18.77
E.8	log(Et.2+1)*week + har	7160.74	-3571.37	9	26.25	6	0.000	0	22.35
E.7	log(Et.2+1)*week	7165.13	-3575.57	7	30.65	7	0.000	0	22.09
E.NULL	~1	7683.42	-3837.71	4	548.94	18	0.000	0	~

Table S2. The table shows the averaged model coefficients from best models with ΔAIC < 6 for Adult, Juvenile and Egg population logged weekly counts. Time lagged stage densities are shown as representing Survival within or Recruitment to the target stage, harA and harJ = 40% harvest of Adults or Juveniles every 7 days and week is a continuous variable. In the table below a colon indicates interaction between two main effects.

	**Adult dynamics**			**Juveniles**	**Egg dynamics**			
**Variables**	Ad.6	Ad.19	Average	J.15	E.14	E.13	E.19	Average
(Intercept)	3.4083	3.8293	3.6188	6.6875	6.9885	6.8546	7.1080	6.9837
Survival	0.4700	0.3908	0.4304	-0.1585	0.2047	0.2047	0.1764	0.1952
week	0.0055	0.0060	0.0058	-0.0127	0.0128	0.0127	0.0082	0.0112
harA	0.5177	-0.1040	0.2069	-0.1787	-0.1874		-0.3209	-0.2542
harJ	0.4604	-0.0895	0.1854	-0.0297	-0.2170		-0.4490	-0.3330
Recruitment	-0.0998	-0.0975	-0.0986	0.1674	-0.4589	-0.4591	-0.4584	-0.4588
Survival:week	0.0000	-0.0001	0.0000	0.0019	-0.0008	-0.0008	0.0002	-0.0005
Survival:harA	-0.1177		-0.1177	0.0054			0.0139	0.0139
Survival:harJ	-0.1036		-0.1036	-0.0322			0.0702	0.0702
week:harA	0.0029	0.0018	0.0023	0.0031			0.0074	0.0074
week:harJ	-0.0076	-0.0061	-0.0069	0.0117			0.0059	0.0059
week:Recruitment	-0.0006	-0.0006	-0.0006	0.0003	-0.0014	-0.0014	-0.0015	-0.0014
harA:Recruitment	-0.0010	-0.0039	-0.0024	0.0064				
harJ:Recruitment	0.0054	0.0026	0.0040	0.0275				
Survival:week:harA	-0.0003		-0.0003	0.0002			-0.0011	-0.0011
Survival:week:harJ	0.0002		0.0002	-0.0003			-0.0016	-0.0016
week:harA:Recruitment	-0.0003	-0.0004	-0.0003	-0.0003				
week:harJ:Recruitment	0.0007	0.0007	0.0007	-0.0020				
**Weight**	**0.522**	**0.477**		**0.998**	**0.816**	**0.087**	**0.066**	

(b) Multivariate Analysis of Variance of Harvest treatments on Post-Harvesting Phenotype

To determine the role of harvesting on the final phenotypes in either high or low food juvenile growth environments we used MANOVA to model log age-at-maturity and log size-at-maturity as a function of the factorial harvest treatment (har =Unharvested, Juvenile or Adult Harvested) and food availability (Low or High). In addition we included *a priori* defined measures of density to control for differences between replicates in competition-related food availability as numbers change. These include total survival (surv), the summed daily densities (i.e. weighted density =densWt) and the median density (densmed) as a consequence of evolved changes in growth rates. Each of these juvenile density covariates can influence adult phenotype in a unique way and could be a consequence of any evolved changes in investment in life-history as well as differences between genotypes from each family selected for the life-history assay (see life-history assay methods). To control, as would be done in the analysis of a randomised block design, for the random differences between populations (pop) within treatment (har) and random difference between families (fam) within populations these nested factorial covariates were included as fixed effects (whether significant or not). To generate **Figure 2e** the covariates for survival, weighted density and median density were standardised with the same mean values across all treatments at the last timepoint. The interaction between harvest treatments and food availability on the phenotype is significant (Table S1) and therefore our full model is our minimal model:

(log(age), log(size))~harvest*food + survival + densWt + densmed+ survival:densWt+ meddens:densWt + har/pop/fam

A separate MANOVA model was built for each time point (not shown) and model predictions of age and size for each timepoint are given in table S4.

Table S2. The table gives a transformation of the test statistic which has approximately an F distribution. We used the default test statistic in anova.mlm in R2.10.0, Pillai–Bartlett as it is conservative, being robust to heteroscedasticity between groups (see anova.mlm help files).). See text above for description of variable names.

MANOVA term	Df	Pillai	approxF	numDf	denDf	Pr(>F)
(Intercept)	1	0.99964	45445	2	33	< 2.2e-16
Har	2	0.66006	8	4	68	1.49E-05
Food	1	0.99475	3129	2	33	< 2.2e-16
Surv	1	0.61226	26	2	33	1.63E-07
densWt	1	0.81466	73	2	33	8.35E-13
Densmed	1	0.06218	1	2	33	0.346721
har:food	2	0.32963	3	4	68	0.014468
har:pop	3	0.36826	3	6	68	0.027127
surv:densWt	1	0.14809	3	2	33	0.071046
densWt:densmed	1	0.24639	5	2	33	0.009396
har:pop:fam	36	0.93657	1	72	68	0.779319
Residuals	34					

Table S3. The Tables give a test statistic, F, for the effect of each manipulated term, density covariate while controlling for the nested experimental design on both (**a**) Age at Maturity and (**b**) Size at Maturity from the minimum adequate MANOVA model (i.e. Table S1). See text above for description of variable names.

(a) LOG (Age):	Df	Sum Sq	Mean Sq	F value	Pr(>F)	
har	2	0.354	0.177	32.928	1.11E-08	***
food	1	32.255	32.255 5	993.371	< 2.2e-16	***
surv	1	0.274	0.274	50.825	3.07E-08	***
densWt	1	0.798	0.798	148.285	5.99E-14	***
densmed	1	0.011	0.011	2.122	0.154	
har:food	2	0.016	0.008	1.475	0.242	
har:pop	3	0.098	0.033	6.045	0.002	**
surv:densWt	1	0.032	0.032	5.909	0.02	*
densWt:densmed	1	0.058	0.058	10.743	0.002	**
har:pop:fam	36	0.189	0.005	0.977	0.528	
Residuals	34	0.183	0.005			

(b) LOG (Size)	Df	Sum Sq	Mean Sq	F value	Pr(>F)	
har	2	0.00088	0.00044	0.078	0.924	
food	1	2.08668	2.08668	374.468	< 2.2e-16	***
surv	1	0.01273	0.01273	2.284	0.139	
densWt	1	0.00331	0.00331	0.593	0.446	
densmed	1	0.00089	0.00089	0.159	0.692	
har:food	2	0.06689	0.03344	6.001	0.005	**
har:pop	3	0.00428	0.00143	0.255	0.856	
surv:densWt	1	0.00003	0.00003	0.005	0.942	
densWt:densmed	1	0.00268	0.00268	0.48	0.492	
har:pop:fam	36	0.14171	0.00394	0.706	0.846	
Residuals	34	0.18946	0.00557			

Table S4. The table estimates the mean coefficient/effect size of each significant term in the minimal MANOVA model for log(age) and log(size) at maturity. See text above for description of variable names.

**Terms:**	**Har**	**food**	**Surv**	**densWt**	**densmed**	**har:food**	**surv: densWt**	**densmed: densWt**
**log(age)**	0.35443	32.25509	0.27353	0.79804	0.01142	0.01589	0.03181	0.05782
**log(size)**	0.00088	2.08668	0.01273	0.00331	0.00089	0.06689	0.00003	0.00268
**Df**	2	1	1	1	1	2	1	1

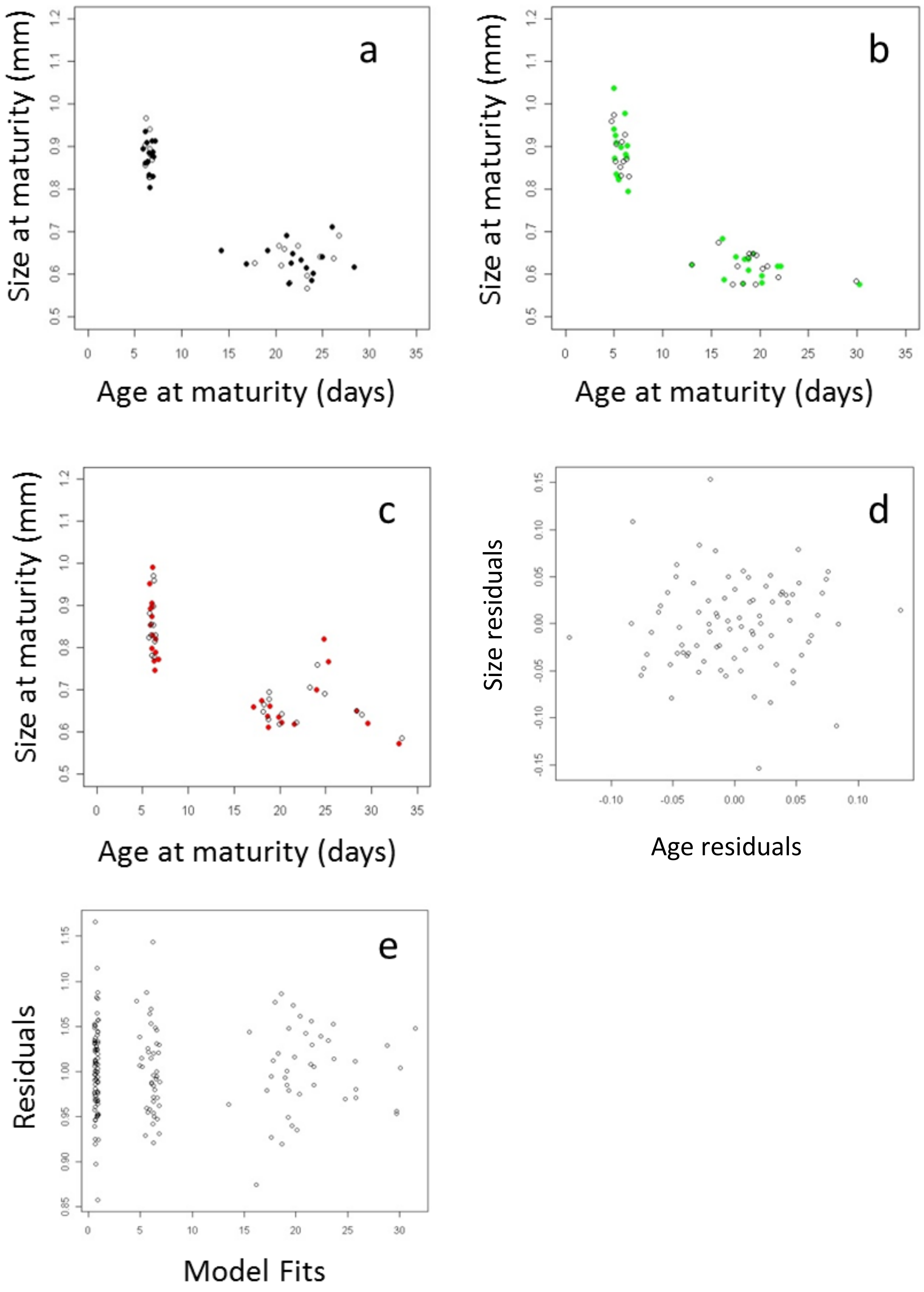

Figure S2. Panel plot summarising goodness-of-fit of the minimal manova of the post-harvesting phenotype at the end of the experiment where we compare model fits (open circles) with observed family means of age and size at maturity (closed circles) for (a) unharvested (mean difference between paired observed and fits is zero; age/size: t= -0.1177/0.0069, df=27, p>0.90), (b) juvenile harvested (age/size: t= -0.1153/-0.1198, df=27, p>0.90) and (c) adult harvested treatments (age/size: t= -0.1368/-0.0168, df=27, p>0.87) when each density covariate are kept at observed replicate means for each food availability. Model residuals for age and size are distributed evenly (d) and a plot of residuals vs fitted values suggests the distribution is relatively constant with a change in mean.

Table S5. The table displays the age and size at maturity of female mites from each life-history assay time point either as measured family means or MANOVA predicts controlling for differences in survival between tubes within assays (see explanation at 2a above) or . A separate model, as described above, was built for each assay time point. For assay time point 1, where covariate information for the median and weighted density are unavailable, mid points between assay time point 0 and 2 are used to make best estimates. We have shown our MANOVA is a good predictor of the original data (see Figure S2). See Figure 2 to understand variation between families at timepoint 4. WT ´= wild type, U = unharvested, J = juvenile harvested, A = adult harvested.

		Predictions using tube means				Predictions using treatment means				Original treatment means			
		Age (days)		Size (mm)		Age		Size		Age		Size	
Time	Harvest	Low	High	Low	High	Low	High	Low	High	Low	High	Low	High
Timepoint 0	WT	12,52	3,88	0,75	0,89	12,23	3,85	0,68	0,89	12,32	3,81	0,63	0,88
	U	14,64	6,05	0,82	1,08	14,64	6,05	0,82	1,08	11,24	5,06	0,64	0,94
Timepoint 1	J	14,57	5,79	0,83	1,07	14,57	5,79	0,83	1,07	11,85	6,01	0,65	0,94
(week 18)	A	14,29	5,93	0,83	1,06	14,29	5,93	0,83	1,06	12,50	5,06	0,65	0,98
	U	14,11	6,63	0,68	0,90	14,31	6,62	0,68	0,90	14,12	6,69	0,69	0,91
Timepoint 2	J	15,01	6,02	0,69	0,89	14,16	6,05	0,69	0,89	15,06	6,01	0,69	0,89
(week 37)	A	13,36	6,47	0,70	0,87	13,54	6,38	0,70	0,87	13,45	6,43	0,70	0,87
	U	15,58	5,28	0,65	0,87	16,51	5,28	0,63	0,87	15,67	5,30	0,65	0,88
Timepoint 3	J	16,56	5,37	0,67	0,83	16,53	5,30	0,67	0,84	17,62	5,24	0,65	0,85
(week 67)	A	16,84	5,32	0,65	0,92	16,13	5,35	0,65	0,92	16,10	5,44	0,66	0,90
	U	21,94	6,57	0,63	0,88	21,99	6,54	0,63	0,88	22,10	6,56	0,64	0,88
Timepoint 4	J	19,24	5,54	0,62	0,89	20,41	5,52	0,62	0,89	19,37	5,61	0,62	0,89
(week 95)	A	22,42	6,19	0,66	0,85	21,67	6,33	0,67	0,85	22,72	6,14	0,66	0,85

(c) Supporting Information for genetic analyses

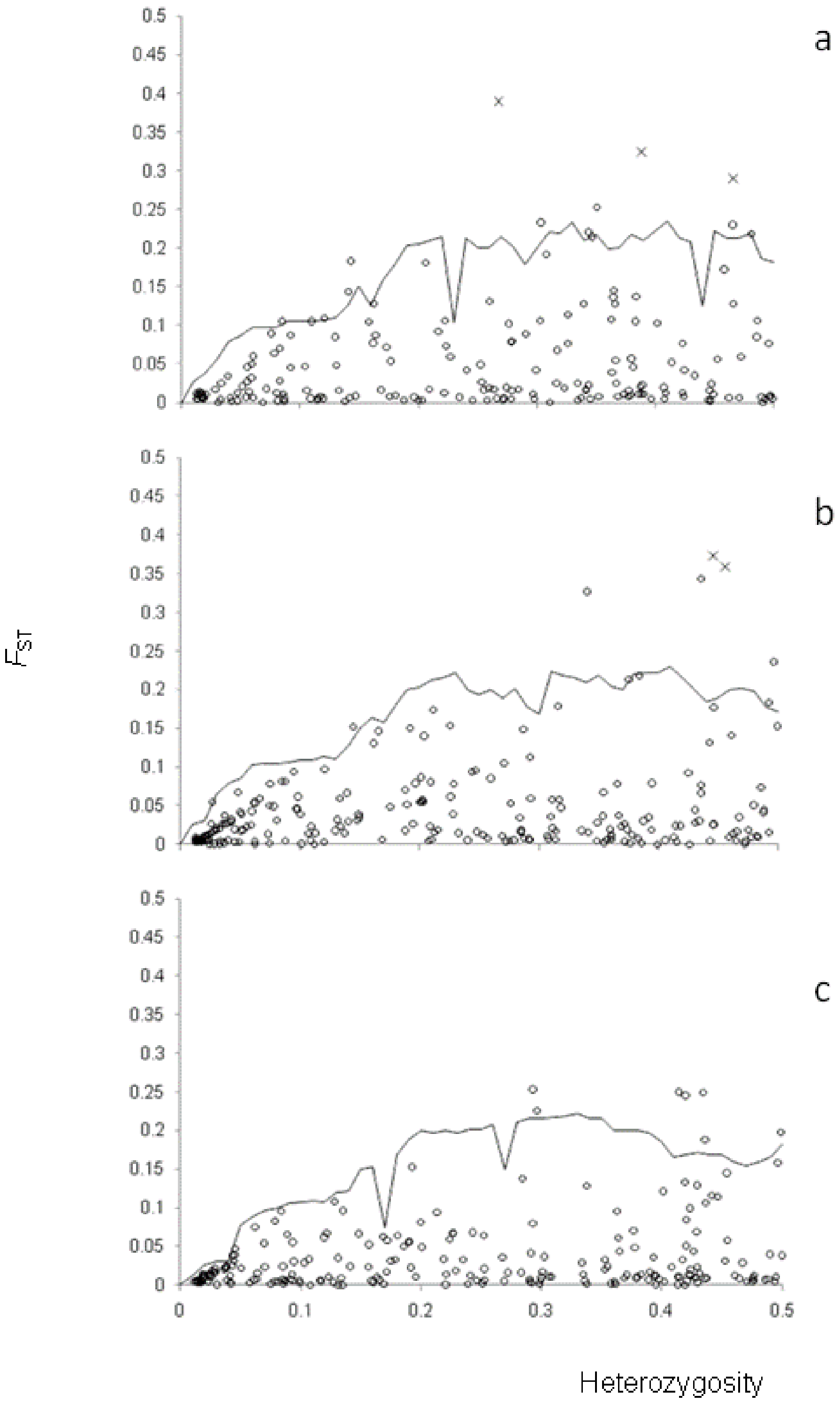

Figure S3 Outlier analysis of genetic divergence for individual AFLP loci calculated between week 0 and week 37 under **(a)** adult harvesting, **(b)** juvenile harvesting and, **(c)** no harvesting. Open circles represent neutral loci falling below the 0.995 quantile value (solid line), whereas candidate loci putatively under divergent selection are open circles above the quantile line, or crosses if the locus was identified as an outlier in more than one population replicate.

(d) Calculation of R_0 values from the life-history data

To understand the feedback from adaptation to the dominant local environmental conditions, strong resource competition/ starvation, on population dynamics we calculated the **basic reproductive rate** (**R_0**) based on the family mean life-history trait values for age-at-maturity, size-at-maturity and daily survival rates per harvest treatment from our low food phenotype full sib females (See life-history assay methods in SI and Box 1). Low food phenotypes are chosen as they better represent the food limited conditions experienced in the microcosm tubes. Estimates of fecundity were obtained from the regressions in the main text based on the results of an earlier experiment (Plaistow *et al.* 2006). We worked with peak fecundity produced between, and including, day 2 to 7 post maturity and not fecundity per lifetime. The effects of size on fecundity under low food conditions is not as pronounced as under high food conditions and is dependent on adult survival. The effects of size on fecundity measured up to 8 days post maturation is neutral, but peak daily fecundity in days 2-4 post maturation is strongly linked to increased size. Therefore adult harvested phenotypes are under strong selection to invest in increased body size where unharvested phenotypes are not. Fecundity estimates for unharvested and juvenile used the neutral size-fecundity relationship whereas for adult harvested phenotypes the relationship between size and fecundity 2-4 days post maturation was used.

Where $R_0 = (l_x * m_x)$

Where R_0 is the sum of l_x, the chance of an individual surviving to age x, times m_x, the number of offspring produced during the time from age x-1 to x. Due to the overlapping generations we correct R_0 as follows:

$R_0 = \exp((\ln R_0)/T_c.)$

R_0 is corrected by T_c, the **average cohort lifespan,** which is the average time from the birth of an individual to the birth of one of its offspring. As the average time from maturity to peak fecundity is 3 days we took T_c to be the average time to sexual maturity plus 3 days.

We created a function to calculate R_0 in R2.14.1 and using 1000 resamples from the family mean life-history data combined with resamples of fecundity coefficients (selected from a normal distribution with the same mean and sd as our data) we estimated bootstrapped mean and confidence intervals of R_0 per assay time point per harvest treatment.

This analysis was undertaken to show how a suboptimal phenotype with low R_0 has changed during the study and changed a declining population into an increasing one. But also to show how differences in the life-history over time and between treatments manifest themselves in differences in population dynamics.

We compared our estimates of R_0 from life history data with the average population growth rates across all replicates per treatment. We expect high correlation between estimates of R_0 and population growth rates as R_0 per generation, corrected for generation time or not, corresponds to other measures of population growth rate such as λ (Caswell 2000, p128-129).

4. SI References not in main text

Beckerman A., Benton T.G., Ranta E., Kaitala V. & Lundberg P. (2002). Population dynamic consequences of delayed life-history effects. *Trends in Ecology & Evolution*, 17, 263-269.

Beckerman A.P., Benton T.G., Lapsley C.T. & Koesters N. (2003). Talkin' 'bout my generation: Environmental variability and cohort effects. American Naturalist, 162, 754-767.

Beckerman A.P., Benton T.G., Lapsley C.T. & Koesters N. (2006). How effective are maternal effects at having effects? *Proceedings Of The Royal Society B-Biological Sciences*, 273, 485-493.

Benton T.G., Lapsley C.T. & Beckerman A.P. (2001). Population synchrony and environmental variation: an experimental demonstration. *Ecology Letters*, 4, 236-243.

Caswell, H. (2000). Matrix population models: construction, analysis, and interpretation. 2nd Ed. Sinauer Publishing.

Plaistow S.J., St Clair J.J.H., Grant J. & Benton T.G. (2007). How to put all your eggs in one basket: Empirical patterns of offspring provisioning throughout a mother's lifetime. *American Naturalist*, 170, 520-529.
